# Participatory Action Research as a Framework for Transdisciplinary Collaboration: A Pilot Study on Healthy, Sustainable, Low‐Income Housing in Delhi, India

**DOI:** 10.1002/gch2.201800054

**Published:** 2018-11-13

**Authors:** Emily Nix, Jacob Paulose, Clive Shrubsole, Hector Altamirano‐Medina, Kristine Belesova, Michael Davies, Renu Khosla, Paul Wilkinson

**Affiliations:** ^1^ UCL Institute for Environmental Design and Engineering Bartlett School of Environment, Energy and Resources University College London Central House, 14 Upper Woburn Place London WC1H 0NN UK; ^2^ Centre for Urban and Regional Excellence 4, Second Floor, Zamrudpur Commercial Complex, Greater Kailash New Delhi 110048 India; ^3^ Air Quality and Public Health Group Environmental Hazards and Emergencies Department Centre for Radiation Chemical and Environmental Hazards Public Health England Harwell Science and Innovation Campus Chilton Oxon OX11 0RQ UK; ^4^ Department of Social and Environmental Health Research London School of Hygiene and Tropical Medicine Keppel Street London WC1E 7HT UK

**Keywords:** health, low‐income housing, participatory action research, sustainable development, transdisciplinary

## Abstract

To tackle global challenges, research collaborations need to integrate multiple disciplinary perspectives and connect with local practices to find solutions that are sustainable and impactful. This paper discusses how participatory action research (PAR) is used as a framework for transdisciplinary collaboration to integrate different disciplines and identify healthy and sustainable housing solutions appropriate for local development practices and policy. By analyzing a transdisciplinary research collaboration investigating housing interventions for low‐income settlements in Delhi, reflections and recommendations are provided for other projects wishing to use a similar methodology. It is found that the PAR framework has successfully guided the integration of contrasting methods and improved the impact of research outcomes, resulting in the emergence of new shared practices. However, it proves to be challenging and requires heightened communication and engagement to achieve understanding between all disciplines and practices. It is recommended that focus is given to developing relationships and effective communication channels and that time should be preallocated for reflection. The work provides insights for integrating academic disciplines, the community, and relevant stakeholders in the cocreation of evidence that is paramount to formulate effective solutions to global challenges.

## 1 Introduction

Today's global challenges are complex, requiring the involvement of multiple sectors to develop multifaceted solutions that support sustainable development. Furthermore, it is considered that to tackle these global challenges and inequalities, “projects must evolve consistently into on‐going programs and long‐term links/alliances must be established for the provision of lasting support to communities.”^1^ Within research, this calls for cutting‐edge collaborations connecting academic theory to local practices and policies. Transdisciplinary approaches focus on integrating academic disciplines with nonacademic or experiential knowledge through collaboration with stakeholders from outside academia.^2^ Moreover, it is recognized that “transdisciplinary collaboration is reliably a more efficient way of tackling some of the most pertinacious challenges.”^3^ Thus to tackle global challenges, scientific disciplines need to transcend their traditional boundaries and allow the integration of multiple disciplinary perspectives, methodologies, and methods as well as relevant stakeholders to find solutions that are effective and impactful. As research collaborations transition to use transdisciplinary approaches, relevant guidance is essential to support these collaborations and ensure best practice outcomes.

In this context, this paper discusses the use of participatory action research (PAR) as a framework for transdisciplinary collaboration to develop and assess strategies for healthy, sustainable housing in participation with residents from an informal settlement in Delhi, India, as part of the “Optihouse project.” We explain the pertinence of PAR as a framework to facilitate transdisciplinary collaboration and employ mixed‐methods research that links theory to practice, and describe its application in our study setting. We conclude by providing reflections on our experience of the use of PAR and outline key recommendations for future research and researchers wishing to employ a similar approach.

## 2 Optihouse Project

The Optihouse project is a transdisciplinary collaboration between academic actors (from epidemiology, building science, architecture, social and environmental sciences), the community (residents from the settlement), and development practitioners (local Non‐Governmental Organization (NGO)). The research aimed to develop and apply methods to improve the design, refurbishment, and operation of housing for low‐income households, which is low cost, low in environmental impact, high in performance, improves quality of life and wellbeing and considers local constraints and desires. It is a small‐scale pilot project focused on three international settings. In this paper, we describe the processes relating to our research site in Delhi.

### 2.1 Research Background

Decent housing is fundamental for good health and well‐being^4^, ^5^, ^6^, ^7^ and for meeting sustainability objectives.^8^ Decent housing should protect against the elements (weather), have a safe structure, be free of hazards (pests, disease vectors), provide adequate facilities (for food preparation, personal hygiene, sleeping), provide a comfortable environment, and offer space for communication and social exchange to promote health.^9^ Yet globally, informal housing is the predominant mode of shelter provision,^10^ this can be particularly seen in the formations of slums in developing countries.^11^ These settlements are unhealthy places with high risks of infection and injury,^12^ due to inadequate housing quality and a lack of basic services. There is limited research focused on identifying solutions and pathways to achieve improved housing in low countries, especially in the context of slums,^13^ where significant benefits are expected.^9^ Given the scale of inadequate housing provision and failure of housing policies,^14^ it is vital to understand how informal development practices could be enhanced to achieve health, sustainability housing for low‐income populations. Thus, this calls for the joint involvement of multiple academic disciplines and local residents in the research process.

### 2.2 Methodology

PAR was selected to develop a suitable study design for this project to integrate the different research and residents perspectives. “PAR provides a methodology… …to guide community interventions, address issues of injustice, and engage in research that increases knowledge and changes the actual conditions of communities.”^15^ PAR is action orientated, participatory, and systemic in its approach.^16^ It allows a dialogue between theory and practice^17^ and uses knowledge generated with participants as a resource for participatory decision making to create change.^18^ Participatory approaches are designed to reduce potential misunderstandings and exploitation between researchers and marginalized groups (such as those residing in informal settlements)^19^ and achieve social change and empowerment as a part of the research process.^20^ This process may not only improve the quality of research through providing local and contextual insights but also has important implications for intervention sustainability and appropriateness,^21^ helping to support the translation of theory into local practices.^22^

Following PAR theory, the research team consisted of field facilitators, local researchers (from the Centre for Urban and Regional Excellence (CURE), and researchers from University College London and the London School of Hygiene and Tropical Medicine, all of whom were involved in the design and planning of the research. The field facilitators are local residents from the settlement and participants in the study. There is close interaction between them, other participants, and the local researchers.

### 2.3 Case Study Setting

Delhi has experienced a rapid population growth,^23^ which has led to widespread development of unauthorized and informal settlements, with only 24% of the housing stock planned.^24^ Resettlement (relocation) of households has been the prevalent policy, with few in situ upgrades.^25^ Resettlement colonies generally have been placed on the periphery of the city, with variable services and (empty) plot sizes.^26^

**Figure 1:**
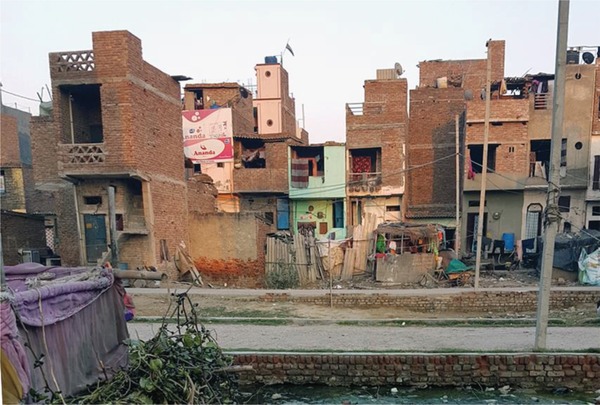
A typical street and the range of dwellings found in the resettlement colony, Savda Ghevra.

The resettlement colony Savda Ghevra, on the northeast edge of Delhi, was selected as a case study to understand how interventions and designs can improve current conditions and build on the already existing incremental processes. The settlement was developed by the Delhi Urban Shelter Improvement Board in 2006 to relocate slum dwellers from inner‐city areas. It currently has around 7000 households (population ≈35 000). Households were provided bare plots of 12.5 or 18 m^2^ for independent construction. The building process is incremental and relies on the families' available skills, economic capabilities, materials, and resources, with little or no external assistance. The resulting housing conditions mostly fall short in terms of health and sustainability.^27^ A typical street and the range of dwellings can be seen in **Figure** **1**.

Households were recruited to participate in the research by community meetings, in total 27 households signed up. It was emphasized that the project required an inclusive sample of participants, representative of the diversity of households present in the resettlement but participation was encouraged by “any interested” community member.

## 3 Research Framework and Activities

In this section, the PAR framework and principles used to support integration of methods are described. We then provide an overview of research activities under this framework.

### 3.1 PAR Framework

**Figure 2:**
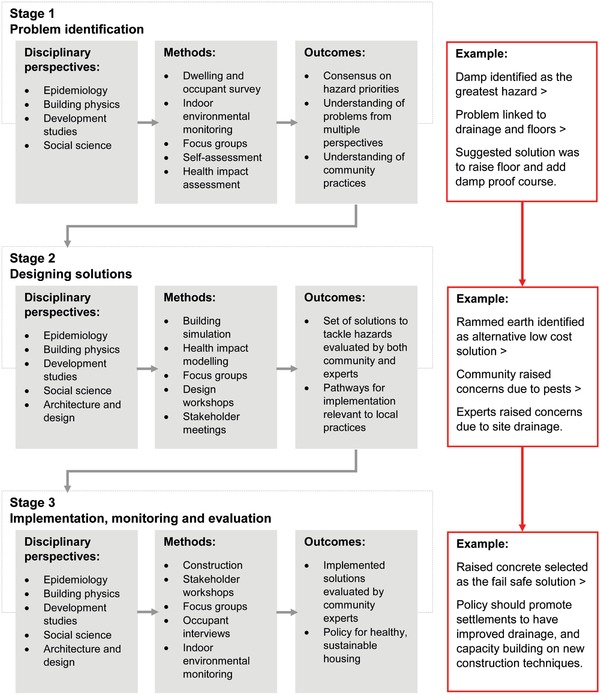
Three‐stage PAR process, integration of disciplinary perspectives and corresponding methods, and enabled outcomes.

The research methodology was guided by a three‐stage PAR framework: problem identification; designing solutions; and implementation, monitoring, and evaluation,^28^ which is typically followed in PAR, with each stage sequentially informing another. We collaboratively designed the research methods for each stage in planning sessions; this permitted the integration of both disciplinary perspectives and resident's perspective on aims, processes, and methods, enhancing the appropriateness and acceptability of ideas of all stakeholders involved. **Figure** **2** provides a visualization of the three‐stage process. Here, the outcomes enabled by the integration of the disciplinary perspectives and their methods are summarized. Due to the circular and reflective nature of PAR, there have been changes and the detailed planning has been on‐going throughout the research process.

### 3.2 Integration of Quantitative Methods

PAR is often associated with a qualitative research approach only and thus it is important to advocate it as a mixed‐methods approach.^29^ A mixed‐methods approach raises questions of ontological and epistemological compatibility,^30^, ^31^, ^32^ such issues also arise from the use of quantitative methods in participatory action research. Marti^29^ carried out a review of how quantitative methods (QUAN) have been integrated within PAR, categorizing them into four approaches under either sequential integration or embedded integration. This categorization can be seen in **Table** **1**. For this work, the same principles were followed to integrate methods and overcome compatibility issues.

**Table 1:** Four ways of integrating quantitative methods in participatory dynamics of action research, adapted from ref. 29

	Sequential integration		Embedded integration	
	QUAN → PAR	PAR → QUAN	QUAN(par)	PAR(quan)
Goals of QUAN within action research process	Improve reflection with evidence‐based data	Monitor changes and provide relevant data for evaluation	Improve deliberation and decision making	Improve observation; engage participants in the process
Role of participants in QUAN	Data receivers	Data receivers	Data producers	Data producers
Action research cycle	Evaluate → reflect	Act → evaluate	Reflect, plan	Observe, act

### 3.3 Research Activities

#### 3.3.1 Problem Identification

**Figure 3:**
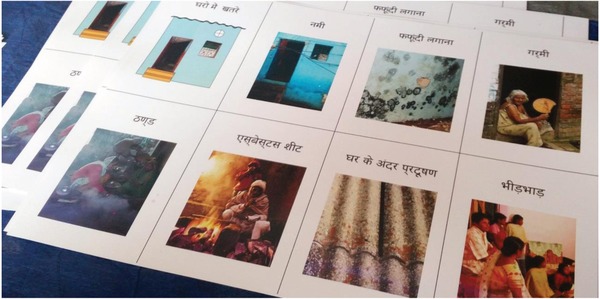
Pictorial hazard cards used by the residents to assess their household hazards.

Health impact assessment methods were used to derive broad quantification of local housing‐related health risks, drawing on data collected from dwelling and occupant surveys, indoor environmental monitoring, and literature of evidence on the underlying health risks. Full architectural drawings were produced for each household and an assessment sheet was completed by the local researchers to record the severity of the hazards. This risk assessment method yielded a semi‐quantitative ranking of the 22 housing hazards identified in the resettlement colony. This ranking was complemented by the local resident's perspective on the health and wellbeing hazards in their dwellings acquired through hazard self‐assessment. The residents were given a set of 22 pictorial hazard cards, each card picturing a potential hazard (e.g., wet walls for damp), as shown in **Figure** **3**. This was followed by a discussion on the meaning of each hazard and a request for residents to rank these in order of importance to them.

The assessment from the researchers revealed that heat, cold, indoor air pollution, mosquitoes, sanitation, and personal hygiene were the most highly ranked hazards in terms of likelihood of occurrence and causing harm; whereas self‐assessment by the participants indicated that damp, mold, heat, mosquitoes, food infestations, and pests were the top prioritized hazards. This revealed significant differences between identified ranked hazards by the residents and the researchers, which indicated that there was a need to understand the differences in the perspectives and build consensus.

Five focus groups with residents grouped by archetype were then held, which were initially planned to discuss solutions to the identified problems. However, on reviewing the differences in the self‐assessment and survey risk‐assessment ranking, we used the focus groups to acquire a better understanding of residents' perspective on housing problems to bridge the gap between the researchers and the residents. We used the quantitative self‐assessment rankings and survey risk‐assessment rankings as evidence for discussion, and further investigated where and when certain hazards occurred, how residents coped with these, and why there was a difference in priorities of hazards from the residents' versus researchers” perspective. This gave us an insight into the daily impact of inadequate housing on residents' everyday practices and hence, health and well‐being. The discussions allowed a consensus to be reached on hazard prioritization, as shown in **Table** **2**. These hazards were the basis of the designs, interventions, and solutions that were developed in the next phase.

**Table 2:** Comparison of hazard priority based on surveying by local researchers and self‐assessment against those agreed upon by both after focus groups

Rank no.	Survey‐based risk assessment	Self‐assessment	Consensus after focus groups
1	Heat	Damp	Damp
2	Cold	Mold	Mold
3	Indoor air pollution	Mosquitoes	Heat
4	Mosquitoes	Pest	Cold
5	Sanitation	Food infestation	Mosquitoes
6	Personal hygiene	Heat	Indoor air pollution

#### 3.3.2 Designing Solutions

Following the problem identification stage, the project entered the solution‐generating phase. For this stage, the research team felt that more architectural expertise was required and involved an experienced architect with prior involvement in the design of low‐income housing. Site walkthroughs, by an interdisciplinary team, allowed understanding of the causality of the prioritized hazards as well as existing local solutions. Time‐transacts of residents' daily household routines were collected to understand their practices, needs, and their relationship with the house. These data provided inspiration, based on community perspective, for the ideation of solutions.

Ideation happened through two iterative cycles; the first cycle focused on exploring options and generating solutions and the second cycle focused on refining ideas, prototyping and testing these prototypes through community meetings. Meetings with external experts allowed exploration of alternate materials and technologies; potential options identified included adobe bricks or rammed earth floor, which could also provide livelihood opportunities. An internal design workshop brought together the different disciplines to share the previous learnings and develop initial design solutions. The developed design ideas were then modeled using building simulation to calculate energy saving and changes in exposure from the identified key hazards (heat, cold, air pollution, and damp/mold). Inputs for a base case model were gathered and the simulation outputs were compared against the monitoring data to calibrate the model. The changes in exposure then informed health impact calculations, to assess health benefits and drawbacks of each option. These quantitative assessments were then used as evidence to discuss during focus groups with resident participants. The factsheets illustrating the different design options with related health and energy benefits were designed in close collaboration with the field facilitators to ensure that the pictorial representations were understandable to the participants. The focus group provided an understanding of the acceptability of alternative materials, techniques, and solutions to residents' practices, needs, and aspirations. A platform was created between the residents and various stakeholders in the form of a participatory workshop, with expert participants working locally in housing, sustainable design, and/or architecture, to develop further design ideas and provide feedback on the findings so far. The local stakeholders found it challenging to develop suitable designs due to the constraints faced by the residents, although this challenge later resulted in bringing some “out of the box” ideas. For instance, one abstract idea of a “house covered with a raincoat” led to the development of a false roof with a garden cloth to increase the thermal comfort during summers. The workshop led to further design options and the establishment of a board of experts to provide oversight over the remaining design, implementation, and monitoring phases and to feed into policy discourse.

**Figure 4:**
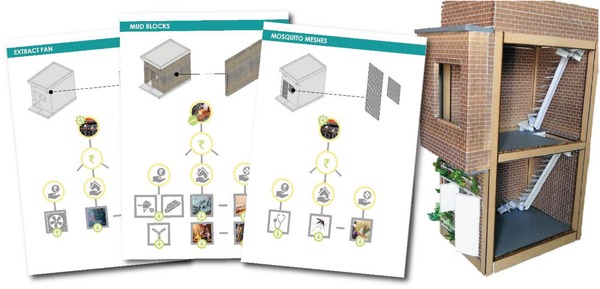
Fact sheets and model prototypes of solutions, as used in community workshops.

A matrix of solutions was compiled by housing elements (such as roof, floor, walls, and ventilation provision) with results of building simulations and health impact calculations. A second expert meeting was held to elicit feedback on each solution and provide guidance for implementation. Based on expert feedback, design/adaptation solutions were further refined before being presented to the participants of the project. The local researchers and field facilitators created physical model prototypes of the solutions and factsheets depicting the materials, and semi‐quantitative assessment of the energy and cost savings and the potential benefits to health, well‐being and productivity, as shown in **Figure** **4**. These provided a visual understanding of how the interventions could be implemented. The community workshops were held to gather feedback on the designs and acceptability of the solutions. At the end of the community workshop, each of the participants ranked the solution in order of preference and their respective reasons were recorded.

#### 3.3.3 Implementation, Monitoring, and Evaluation

**Figure 5:**
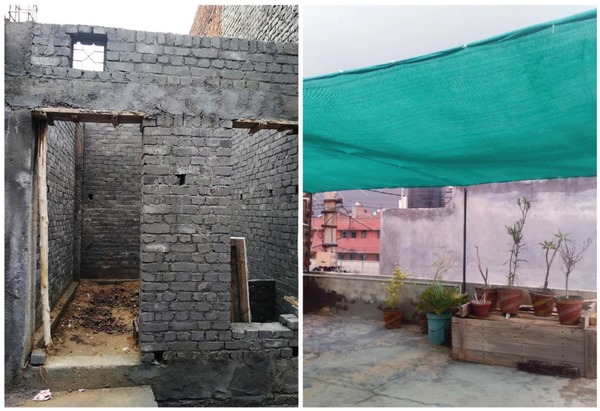
On‐going construction of a newly designed house (left) and implemented solar shading on an existing roof (right).

We are now implementing interventions and new housing designs, as pictured in **Figure** **5**. Full technical drawings and costings of the solutions, which were produced in the designing solutions stage, have been made. We secured further funding to support the households in construction and gaining access to materials. Iterative discussions with the households to determine the intervention desired if any, and the level of external support needed is ongoing. Once construction has finished, we will then monitor the performance of the interventions and designs recording changes in indoor environmental conditions, mosquito counts, and perceived impacts as reported by the occupants. These findings will be fed into focus groups and expert workshops for dissemination and to scale up impact.

## 4 Reflections and Recommendations

In this section, we reflect on the use of PAR as a framework and provide recommendations for others wishing to implement a similar methodology for transdisciplinary collaboration. These reflections and recommendation were generated through a number of collaborative discussions between the research team.

### 4.1 Reflections

The PAR approach provided a framework that enhanced the integration of disciplines and methods and allowed the boundaries of the work and potential changes to be clearly understood. It also enhanced communication between contributing actors while allowing for flexible boundaries. This proved to be very helpful especially in the initial stages of the project and underpinned the communication between the research team and participants throughout the project. Beyond this, the PAR framework supported transdisciplinary collaboration and allowed the following outcomes:

#### 4.1.1 An Enhanced Shared Understanding

The mix of “top‐down” and “bottom‐up” methods used in the initial stage of problem identification (housing surveys, focus groups, visualization cards, etc.) helped to establish shared understanding of the different perspectives and priorities of the research team and local participants, which would not have been achieved without the guiding framework. The circular and reflective nature of PAR allowed a shift in objective of the focus groups and established a clearer understanding of the causes and consequences of housing‐related health hazards. This shift in objective exposed a range of short and long‐term social, economic, physical, institutional, physiological, and environmental impacts due to housing deficiencies as experienced by the residents. It highlighted the underlying problem that poor housing has appreciable negative mental and physical health impacts, which reduces the productivity, and hence residents' income, which in turn affects the quality of food, living, medical health care, recreational activities, and many other aspects of daily living. This affects the development of the economy of the community as a whole. Understanding these realities highlighted the importance of incorporating the participants' insight and the need to develop a common language and terminology grounded in the residents' perspectives to gain a holistic picture. However, this also presented unexpected challenges to the research team in formulating plans of action due to the complexities of the situation.

#### 4.1.2 Expanded Disciplinary Boundaries and Cogeneration of Theory

The direct link to participants expanded disciplinary boundaries as emerging findings needed to be translated to an understandable form for effective communication with the community. The participatory tools and techniques were useful for illustrating the principles of healthy housing development to the reality of participants' lives. This required creative thinking and more communication between team members, which in turn strengthened the relationships and led to an enhanced understanding of the various disciplines' practices and their methods. It also ensured that the methods and outputs were relevant to the realities of people's lives, which increased the relevance of the research, overcame culture difficulties, and thus led to plans that are more realistic. An example was the need to be able to demonstrate the value of any housing adaptations in reducing the costs of healthcare and increasing productivity as a motivation for implementing the adaptation by individual households. This dynamic exchange between the disciplines and participants helped in the cogeneration of theory and allowed the integration of the theoretical perspective of each discipline with the participants' perspectives, instead of imposing pre‐existing disciplinary theories that may be in tension with each other and the community's need.

#### 4.1.3 Enabled the Adaptability and Compatibility of Varying Methods

As discussed above, different types of integration were used at different times through the project to support unified research processes oriented toward an agreed end. For example, in the “designing solutions” phase, the building simulation and health impact calculations were carried out sequentially before being used as evidence into focus groups and expert workshops. In the focus groups and expert workshops, embedded integration was used to rank hazards and design solutions using a multicriteria decision analysis matrix to quantify perspectives. These processes allowed flexibility in the presentation of various sources of evidence and helped achieve understanding amongst the various stakeholders. Integrating methods in this way also helped to resolve possible incompatibility of epistemology and ontological disciplinary differences and capitalized on the strengths of multiple disciplinary domains.

#### 4.1.4 Increased Impact and Sustainability of Research Outcomes

The reflective nature of PAR can sometimes be slow in progression but, as evident from our experience in this project, it allows analysis of a problem and the formulation and implementation of solutions to be better informed, more responsive and better suited to the practical challenges and perspectives of the end‐users. The ability to be reactive to changes and incorporate different perspectives allows new opportunities and pathways to be explored. For example, solutions that could result in livelihood opportunities are now being explored after discussions with participants and experts. However, the iterative processes present a challenge to the linear thinking of traditional disciplines and require a greater degree of discussion and exchange with the communities. For example, in working with the participants to implement changes in their homes, agreement on exactly which forms of adaptation to implement was more complex because of the need to reconcile principles of healthy housing with participants' needs, desires, and limitations. Traditionally, epidemiologists would typically define an intervention, intervene, and then measure the changes against controls, as commonly carried out in clinical trials. The project's approach, in which the participants are in control may be nonlinear and time consuming and somewhat at odds with traditional research approaches and values of validity and generalizability. But, we argue that the undefined outputs, in fact, provide enhanced learning opportunities, whereby full understanding of the context of practice can be achieved resulting in increased impact of the research. Without the flexibility to adapt, it would not have been possible to bring all the stakeholders to a common understanding regarding the project as a whole. In the entire process, evaluation reflection and feeding back into the designed process and having the flexibility to accommodate the changes to achieve the final goals unilaterally proved to be very important.

### 4.2 Recommendations

Although PAR allows the integration of different disciplines and linking them with practices on the ground, this project faced a number of challenges. Successful implementation of the PAR approach and transdisciplinary collaboration needs to be explicitly and transparently built into the project design, planning, management, and communication strategies. Below are the key recommendations for other projects that may wish to use a similar approach.

#### 4.2.1 Begin with a “Collaboration Workshop” and Team Field Visit

A workshop and field visit should be held at an early stage in order to share insights, develop interdisciplinary understandings and understand the realities on the ground. This is fundamental to establish a good grounding for the work and ensures that each group and their approach is equally represented. Part of this workshop should include defining what is considered to be a “successful” project and agreeing on short‐term and long‐term objectives as well as the roles and responsibilities of each group. Such activities should be held regularly throughout the project. The commitment to build and maintain relationships is critical to the project success.

#### 4.2.2 Develop an Effective Communication Framework

Effective communication is, of course, a vital step in the challenging process of transdisciplinary integration. However, such challenges can be addressed through the development of a framework to guide the development of shared understanding. Elements of a framework could include reflection on the implicit disciplinary assumptions, vocabularies, cultural values, and norms. Similar reflective practices should be explicitly applied in the process of integration of insights generated as a part of the research process. It should recognize that the research should inform the practitioner as much as the researcher and there should be a continual dialogue to ensure the information flow is two way.

The issues of control and power of the research agenda are difficult to manage at times and require a significant amount of discourse between the research team to arrive at a common understanding. This requires levels of engagement and openness beyond that of traditional research projects. Meetings should be held frequently and significant informal communication should be expected. This can be problematic over different time zones and with differing priorities; however, modern communication technology can aid this process.

#### 4.2.3 Be Prepared for Flexibility

PAR, by nature, is an evolving process and the team needs to be prepared to be flexible and, to be true to the methodology, guided control needs to be given to the participants. This is somewhat problematic and can mean that the project is hard to manage and feels a little chaotic at times. Thus, it is useful to have someone who is there to remind the team of the overarching objectives and ask the participants how they think the work should be taken forward. Further to this, the project should not begin with preconceived ideas but be open instead to accommodate the ideas and inputs customizable to the beneficiaries, location, and so forth. Working in this way places increased need for funding sources to allow a more flexible approach as the project develops. The funding requirements need to be assessed realistically at the proposal stage and adequate levels of contingency built in. The resources should be reflected on throughout the project and made transparent to all participants and researchers.

#### 4.2.4 Build in Dedicated Time for Reflection and to Assess Validity

Such an approach requires a framework that will not just allow for but will positively encourage time for reflection—time and resources at the planning and ongoing stages of the project should be allocated explicitly to this end. This is vital due to the nonlinear processes of PAR, without time dedicated for reflection; it would not be possible to ensure that everyone is on‐board with the current plans and has a full understanding of the findings to date. As part of the reflection process, there should be a place for critical discussion on the research processes and the creation of knowledge and evidence. A framework to assess the validity of the research from the perspectives of all disciplines should be developed at an early stage and this should be used to ensure the work is of high quality and remains true to the transdisciplinary approach. Without a framework and time for reflection, there is a risk of producing findings, which are of poor quality.

#### 4.2.5 Provide Dedicated Training and Support to Researchers

The members of the team should be provided with adequate training and support that recognizes the particular skill set required to work in a transdisciplinary manner. This requires significantly more knowledge of different research disciplines, approaches, and methods. Furthermore, the use of participatory techniques and tools requires knowledge and practice in this area. This requires a significant amount to flexibility from the researchers to draw upon different resources and knowledge while ensuring high‐quality research. Further to this, methods used traditionally might need expanding on, which requires additional time and resources by the research team—this time should be accounted for in the project planning.

## 5 Conclusions

Global challenges are increasingly interdisciplinary, complex, and dynamic, and therefore, require integration of methodological approaches and disciplines to find solutions that are sustainable and impactful. We discussed the use of participatory action research as a framework to integrate different disciplinary approaches and provide a dialogue between theory and practice; we then presented an application to develop solutions for achieving healthy, sustainable housing in a low‐income settlement in Delhi, India. While PAR provided methodological guidance that improved the integration of contrasting methods and improved the impact of the research outcomes, it proved to be challenging at times, requiring a significant amount of communication and engagement to achieve understanding between all disciplines and practices. The direct link with the participants supported the cocreation of knowledge and helped to expanded disciplinary boundaries as the need to communicate in understandable forms pushed methods beyond traditional approaches. This, in turn, helped to improve the interaction between different disciplines and practices, enhancing understanding and integration. The knowledge and the interventions developed were relevant to the local practices, ensuring the sustainability of solutions.

We recommend that researchers wishing to use PAR in a similar approach ensure that focus is given to developing relationships through collaborative workshops and effective communication channels and that the objectives are reviewed collaboratively throughout the project. The process needs to be designed with time for reflectivity and the capacity for flexibility, thus ensuring it is possible to adapt to changes and align the outcomes with all stakeholders. This was a pilot study and so to an even greater extent than normal, we were developing our understanding as we were going. Nevertheless, valuable learning has taken place and we will use this to modify our approach to ongoing and future projects. We hope that the recommendations above may also prove to be useful for other teams. We conclude that the biggest determinant of collaboration success was the extent of engagement between different actors and their emergence into different practices. This work highlights that integration between academic disciplines, the community, and stakeholders in the cocreation of evidence is paramount to formulate effective solutions to that can help tackle today's global challenges.

## Conflict of Interest

The authors declare no conflict of interest.
